# French guidelines for restrictive episiotomy during instrumental delivery were not followed by an increase in obstetric anal sphincter injury

**DOI:** 10.1038/s41598-022-10379-6

**Published:** 2022-04-15

**Authors:** Bertrand Gachon, Xavier Fritel, Olivier Rivière, Bruno Pereira, Françoise Vendittelli

**Affiliations:** 1 Service de Gynécologie Obstétrique et Médecine de la Reproduction, Department of Obstetrics & Gynecology, Centre Hospitalier Universitaire de Poitiers, 2 rue de la Miletrie, 86000 Poitiers, France; 2 INSERM CIC61402, Université de Poitiers, CHU de Poitiers, Poitiers, France; 3 Audipog, Université Claude Bernard Lyon 1-Laennec, Lyon, France; 4 Centre Hospitalier Universitaire de Clermont-Ferrand, 63000 Clermont-Ferrand, France; 5 Institut Pascal, CHU, CNRS, Clermont Auvergne INP, Université Clermont Auvergne, 63000 Clermont-Ferrand, France

**Keywords:** Urogenital reproductive disorders, Epidemiology

## Abstract

The objective was to assess the influence of the French guidelines in favor of a restrictive use of episiotomy on both episiotomy and obstetric anal sphincter injury (OASI) rates during instrumental delivery. It was aulticenter study involving 193 maternities between 2000 and 2016. We included women with a singleton pregnancy, with cephalic presentation at 34 weeks of gestation or more who underwent an instrumental delivery. The study period was divided into three phases: 2000–2005 (reference) 2006–2011, and 2012–2016. We calculated the adjusted relative risk (aRR) of episiotomy and OASI and investigated for changes in episiotomy and OASI rates over time by using Prais–Winsten regression. We considered 96,035 deliveries. The episiotomy’s risk was lower in 2006–2011 (69.4%) and 2012–2016 (59.1%) compared to 2000–2005 (81.2%), respectively: aRR 0.93 [0.92–0.95] and 0.89 [0.87–0.90]. The OASI’s risk was higher in 2006–2011 (2.5%) and 2012–2016 (3.1%) compared to 2000–2005, respectively: aRR 1.30 [1.10–1.53]) and 1.57 [1.33–1.85]. However, Prais–Winsten regression showed no difference in the OASI rate during the study period. We observed a massive decrease in episiotomy use and a moderate increase in crude OASI’s rate but multivariate analysis failed to report an association between these outcomes.

## Introduction

Obstetric anal sphincter injury (OASI) is a complication that occurs in 0.4–5% of deliveries^1–3^. It manifests as a superficial injury of the external anal sphincter and, in the worst cases, a tear of the internal anal sphincter and a complete opening of the rectal mucosae (third- and fourth-degree perineal tears)^1,2^. This condition is associated with an increased risk of fecal incontinence, perineal pain, dyspareunia, and sexual dysfunction^1,2,4^. The main risk factor reported in the literature is instrumental delivery in comparison with spontaneous vaginal delivery, especially for nulliparous women^1,2,5^. Such instrumental deliveries are performed for 12.2% of women in France, which represent approximately 96,000 deliveries each year^6^. The use of episiotomy in childbirth has long been debated by both women and obstetricians. An updated Cochrane meta-analysis reported that for spontaneous delivery, a routine policy of episiotomy offers no benefits in preventing OASI^7^. The indications for episiotomy in high-risk situations such as instrumental delivery have also been debated in much detail^8^. A small pilot randomized trial failed to identify any significant preventive effect^9^. Although several retrospective studies, mainly European national cohort studies, have reported that mediolateral episiotomy has a protective effect against OASI^10–12^, these studies appear to have reported a high rate of episiotomy and a high rate of OASI in the few cases without episiotomy, which seem to be not concordant with French practices. In our country, the 2005 national guidelines recommended restricted use of episiotomy during vaginal delivery, including instrumental delivery^13^. In our university, over an 11-year period, the rate of episiotomy usage reduced from 78 to 16% in cases of instrumental delivery and the rate of OASI occurrence increased from 3.9 to 12.7%^12^. Such an important increase in OASI occurrence might be related to the important magnitude of the decrease in episiotomy rate and the massive speed of such a decrease across the time. This phenomenon could only be observed in a monocentric experience but not at a national level with different teams and local guidelines, explaining why these results need to be confirm (or not) by a multicentric study. Additionally, a recent meta-analysis reported that mediolateral episiotomy should be considered in case of vacuum assisted delivery in primiparous women and that some high levels of evidence studies are required in this thematic^14^. A low level of evidence is likely to explain why there are no clear guidelines regarding the indication of episiotomy to prevent OASI during instrumental delivery^1,2,15^. Another limitation for providing clear international guidelines is the opposition between restrictive and extensive use of episiotomy without a clear consensual cut-off for defining a restrictive use. For example, a restrictive use was defined by a rate under 30% of episiotomy in France and 60% in the Jiang et al. Cochrane meta-analysis^7,13^. Our main objective was to assess the influence of the 2005 French guidelines on the episiotomy rate during instrumental delivery in cases recorded in the AUDIPOG (Pediatric Electronic Records Users Association, Obstetrics and Gynecology) French database. Our secondary objectives were to assess the influence of these national guidelines on the rate of OASI during instrumental delivery and the episiotomy rate according to the type of instrument used for delivery.

## Methods

### Description of the AUDIPOG sentinel network

This historical cohort study evaluated deliveries included in the AUDIPOG sentinel network database (https://www.audipog.net). This network, created in 1994, includes individual data on mothers and infants voluntarily contributed by public and private maternity units from every region in France, and the data are subsequently pooled and used for analyses (data available on reasonable request to the corresponding author). Each maternity unit participates for a given period each year chosen by them, which is usually one month, but may even be the entire year. The composition of the maternity units participating each year in the database has varied since 1994, since some units have closed, etc. During the participation period, the hospitals forward all data about women who gave birth at a gestational age of 22 weeks or more or a birth weight of 500 g or more if the gestational age was unknown, and about the newborns. Details of the methodology for data collection have been published in other studies^16–18^. At the time of this study, the database included 1,051,785 pregnancies from 1994 to 2016 from 256 participating maternity units.

### Population

**Figure 1:**
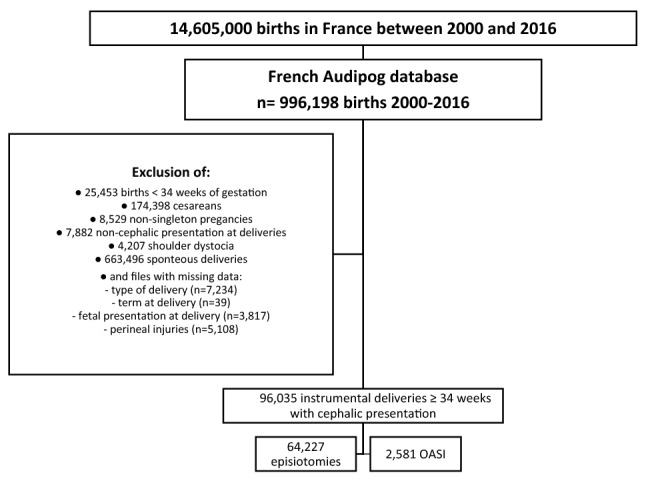
Flow chart.

For this study, we selected deliveries that occurred between 2000 and 2016, which represented 996,198 births from 193 maternity units. All instrumental deliveries of singleton pregnancies in cephalic presentation at 34 weeks of gestation or more were considered. Preterm births at less than 34 weeks of gestation (n = 25,453), cesarean deliveries before or during labor (n = 174,398), non-singleton pregnancies (n = 8529), breech presentations (n = 7882), operative maneuvers for shoulder dystocia (n = 4207), and spontaneous vaginal deliveries (n = 663,496) were excluded. Cases with missing pertinent data for this study were also excluded: no information about the type of delivery (n = 7234), no data about the term at delivery (n = 39), no data for presentation (n = 3817), and no data for perineal trauma (n = 5108). Finally, 96,035 deliveries were included in the study (Fig. 1).

We chose to exclude deliveries complicated by shoulder dystocia because we aimed to focus on the association only between episiotomy rate and OASI occurrence. Shoulder dystocia is associated with OASI occurrence, especially in case of fetal manipulation requirement, whatever an episiotomy was performed or not. It is likely that in cases of shoulder dystocia episiotomy is more often performed to ease fetal manipulations performance and/or to avoid neonatal morbidity than to protect from OASI. This considered we thought that cases of shoulder dystocia could bring confusion in our analysis and chose to exclude them.

### Outcomes

The main outcome was the episiotomy rate. The secondary outcome was OASI occurrence. In the database, episiotomy was registered as a binary variable (yes/no) and perineal tears occurrence as a 5 categorical variables (No/1st degree/2nd degree/3rd degree/4th degree). According to Royal College of Obstetricians and Gynecologists (RCOG) classification, OASI was considered in cases of 3rd and 4th degrees tears^1,2^.

### Statistical analysis

Firstly, the social, demographic, obstetrical, and delivery characteristics of women with an instrumental delivery were compared according to the delivery period (2000–2005 before the guideline publication which occurred in 2005 January vs. two periods following the publication of the guidelines 2006–2011 vs. 2012–2016). Secondly, we compared OASI occurrence according to the study periods, and the episiotomy and OASI rates according to the instrument required for the delivery (vacuum, forceps, spatulas). In December 2005, the French guideline on episiotomies highlighted the need to restrict the use of episiotomy and stressed that each maternity unit should reduce its overall episiotomy rate in vaginal deliveries to less than 30%^13^. These guidelines also reported that the routine use of episiotomy does not protect from OASI and that such a practice is not justified in the case of an instrumental delivery^13^. This justifies the choice of 2000–2005 as the reference period (before the publication of these guidelines).

Categorical variables were compared using χ^2^ tests (or Fischer’s exact test, if applicable) and continuous variables were compared using Student’s t-test. Crude relative risks (RR) of episiotomy and OASI for delivery periods (2000–2005 vs. 2006–2011 vs. 2012–2016) were calculated, with their 95% confidence intervals (95% CI). A log-binominal model was used to adjust for confounding factors or pertinent clinical prognostic factors according to each studied outcome in our work (level of maternity units, parity, and/or head circumference, and/or percentile of birth weight according to gestational age and sex, geographical origin, and/or BMI, and/or type of instrument, and episiotomy for the OASI outcome)^19–21^. If the model failed to converge, we used Poisson regression with a sandwich error term^22^. Adjusted RR (aRR) was calculated using the 95% CI.

To assess the global evolution of the episiotomy and OASI rates, we conducted time-series analyses with a Prais–Winsten regression based on the generalized least-squares method to estimate whether the parameters in a linear regression model within the errors were serially correlated. Specifically, the errors were assumed to follow a first-order autoregressive process. Second, to compare the evolution of the episiotomy and OASI rates according to the studied periods, we performed an autoregressive integrated moving-average (ARIMA) model for time-series, where the disturbances are allowed to follow a linear ARIMA specification, in univariate and multivariate analyses (when independent variables are included in the specification). Adjustment covariates were used for the multivariate analyses. The interaction between year and each covariate was investigated. The results were expressed as adjusted risk ratios (aRR) and 95% CI and were plotted using a forest plot.

As mentioned previously, we excluded cases with missing data for our research (type of delivery, term at delivery, fetal presentation, perineal trauma). We used complete cases for the analysis.

The level of statistical significance was set at p < 0.05. All statistical analyses were performed using the Stata software (V15, StataCorp, College Station, USA).

### Ethical considerations

The Audipog database only contained data collected from medical files in the voluntary maternity hospitals, according to their usual practices without any specific interventions. The present study being a retrospective analysis within this Audipog database, the need of consent is waived by Rhône Alpes Auvergne Regional ethical committee, Grenoble, France IRB 5921; CECIC Rhône Alpes Auvergne, Grenoble IRB 5921. The study, including the previous specific point, was approved by an ethical committee on 26 April 2021 (Rhône Alpes Auvergne Regional ethical committee, Grenoble, France IRB 5921; CECIC Rhône Alpes Auvergne, Grenoble IRB 5921). No core outcome set was implemented for this study or patient involvement in the study design. We received no funding for this study. Methods were carried out in accordance with relevant guidelines and regulations.

## Results

Women’s characteristics of the entire cohort are reported in Tables 1 and 2. The BMI was ≥ 25 for 15.5% of the women and 15.5% was ≥ 35 year old (Table 1). The mean gestational age at delivery was 39.6 [± 1.3] weeks, and the onset of labor was spontaneous for 75.2% of women, while 91.9% received epidural analgesia (Table 2). Among the instrumental deliveries, 40.2% were performed using vacuum, 34.8% using forceps, and 25% using spatulas. The mean birth weight was 3350 [± 449 g], and the cranial circumference was 34.7 [± 1.5] cm (Table 2). Most women gave birth in a level 2 public maternity ward (45.4%).

**Table 1:** Description of social and demographic characteristics of the overall cohort and according to the 3 studied periods.

	Total N = 96,035 % (n)	2000–2005 N = 16,264 % (n)	2006–2011 N = 37,686 % (n)	2012–2016 N = 42,085 % (n)	*p*
**Maternal age**^**1**^	(95,946)	(16,214)	(37,660)	(42,072)	
< 20	2.6 (2453)	3.1 (509)	2.8 (1042)	2.1 (902)	< 10^–4^
≥ 20 to < 35	82.0 (78,643)	83.3 (13,501)	81.7 (30,767)	81.7 (34,375)	
≥ 35	15.5 (14,850)	13.6 (2204)	15.5 (5851)	16.2 (6795)	
**Maternal BMI**^**2**^	(69,689)	(14,542)	(28,722)	(26,425)	
< 18.5	8.9 (6179)	9.4 (1370)	9.0 (2592)	8.4 (2217)	< 10^–4^
≥ 18.5 to < 25	68.6 (47,788)	69.8 (10,146)	69.2 (19,873)	67.2 (17,769)	
≥ 25	22.6 (15,722)	20.8 (3026)	21.8 (6257)	24.4 (6439)	
Family situation (alone)	(55,163)	(11,907)	(20,791)	(22,465)	
	7.5 (4161)	11.0 (1,311)	6.3 (1,317)	6.8 (1,533)	< 10^–4^
**Geographic origin**	(56,063)	(11,142)	(23,438)	(21,483)	
France^3^	71.5 (40,080)	77.3 (8,608)	72.5 (16,996)	67.4 (14,476)	< 10^–4^
South Europe	2.6 (1477)	2.3 (258)	2.5 (583)	3.0 (636)	
North Africa	12.8 (7163)	8.7 (966)	12.4 (2914)	15.3 (3283)	
Africa	3.7 (2065)	2.6 (285)	3.8 (889)	4.1 (891)	
Asia	2.3 (1297)	2.1 (235)	2.2 (521)	2.5 (541)	
Other	7.1 (3981)	7.1 (790)	6.5 (1535)	7.7 (1656)	
Smoking^4^	(77,718)	(15,384)	(30,552)	(31,782)	
	12.3 (9555)	15.2 (2342)	11.7 (3578)	11.4 (3635)	< 10^–4^

For each outcome, the first line reports the number of cases with available data/

^1^In years.

^2^*BMI* body mass index just before the beginning of pregnancy.

^3^France: Continental (metropolitan) France.

^4^Tobacco during pregnancy.

**Table 2:** Description of delivery, neonates, and maternities’ characteristics in the overall cohort and according to the 3 studied periods.

	Total N = 96,035 % (n) Mean (SD)	2000–2005 N = 16,264 % (n) Mean (SD)	2006–2011 N = 37,686 % (n) Mean (SD)	2012–2016 N = 42,085 % (n) Mean (SD)	*p*
**Delivery related characteristics**					
History of C-section	(90,259)	(14,883)	(35,322)	(40,054)	
	7.2 (6506)	6.8 (1019)	7.0 (2474)	7.5 (3013)	0.004
Previous delivery	(91,758)	(15,383)	(35,597)	(40,778)	
0	77.4 (71,010)	77.7 (11,953)	78.2 (27,838)	76.6 (31,219)	< 10^–4^
1	17.5 (16,032)	17.2 (2653)	16.8 (5987)	18.1 (7392)	
> 1	5.1 (4716)	5.1 (777)	5.0 (1772)	5.3 (2167)	
Mean gestational age^1^	(96,035)	(16,264)	(37,686)	(42,085)	
	39.6 (1.3)	39.6 (1.4)	39.6 (1.3)	39.6 (1.3)	0.004
Epidural analgesia	(90,151)	(14,808)	(36,603)	(38,740)	
	91.9 (82,863)	88.1(13,048)	91.4 (33,461)	93.8 (36,354)	< 10^–4^
Pathology during labor^2^	(77,039)	(16,103)	(30,583)	(25,353)	
	59.1(42,603)	44.8 (7,211)	59.2 (18,099)	68.2 (17,293)	< 10^–4^
Mean labor duration^3^	(32,430)	(11,871)	(14,115)	(6,444)	
	3.4 (3.5)	3.3 (3.2)	3.3 (3.8)	4.0 (3.2)	< 10^–4^
Type of instrument	(96,035)	(16,264)	(37,686)	(42,085)	
Forceps	34.8 (33,452)	48.0 (7,799)	35.7 (13,470)	28.9 (12,183)	< 10^–4^
Spatulas	25.0 (23,992)	23.5 (3,825)	23.7 (8,913)	26.7 (11,254)	< 10^–4^
Vacuum	40.2 (38,591)	28.5 (4,640)	40.6 (15,303)	44.3 (18,648)	< 10^–4^
**Neonates related characteristics**					
Birthweight (g)	(95,627)	(16,144)	(37,580)	(41,903)	
	3350 (449)	3369 (452)	3352 (449)	3341 (448)	< 10^–4^
< 2500	2.9 (2792)	2.7 (428)	2.9 (1084)	3.1 (1280)	< 10^–4^
≥ 2500 to < 4000	89.6 (85,694)	89.0 (14,363)	89.6 (33,690)	89.8 (37,641)	
≥ 4000	7.5 (7141)	8.4 (1353)	7.5 (2806)	7.1 (2982)	
LGA > 90th percentile^4^	8.1 (7761)	9.3 (1497)	8.2 (3089)	7.6 (3175)	< 10^–4^
SGA < 10th percentile^5^	9.4 (8953)	9.1 (1475)	9.1 (3430)	9.7 (4048)	0.02
Cranial perimeter (cm)^6^	34.7 (1.5)	34.6 (1.5)	34.7 (1.5)	34.7 (1.5)	0.90
< 34	22.8 (13,305)	22.0 (3233)	22.8 (5418)	23.3 (4654)	0.06
≥ 34 to < 36	50.1 (29,245)	50.5 (7412)	50.3 (11,921)	49.7 (9912)	
≥ 36	27.1 (15,783)	27.5 (4038)	26.9 (6377)	26.9 (5368)	
**Maternities related characteristics**					
Level^7^	(96,035)	(16,264)	(37,686)	(42,085)	
I	14.9 (14,271)	19.8 (3219)	13.8 (5182)	13.9 (5870)	< 10^–4^
II	45.4 (43,599)	41.7 (6774)	46.9 (17,689)	45.5 (19,136)	
III	39.7 (38,165)	38.6 (6271)	39.3 (14,815)	40.6 (17,079)	
Type of maternity unit^7^	(96,035)	(16,264)	(37,686)	(42,085)	
Public	71.3 (68,438)	73.3 (11,915)	72.7 (27,412)	69.2 (29,111)	< 10^–4^
Private	28.7 (27,597)	26.7 (4349)	27.3 (10,274)	30.8 (12,974)	

For each outcome, the first line reports the number of cases with available data.

^1^At delivery (in weeks).

^2^Problem during labor (fetal-pelvic disproportion, fetal heart rate anomaly, dynamic dystocia, etc.).

^3^From 5 cm to full dilatation (in hours).

^4^Large for gestational age at birth (defined by French Audipog physicians as > 90th percentile for gestational age and sex).

^5^Small for gestational age at birth (defined by French Audipog physicians as < 10th percentile for gestational age and sex).

^6^Measure immediately after the birth, in cm.

^7^At birth. Level of maternity ward. Level I: no neonatal department; Level II: presence of a neonatal department and special care in same building or immediate proximity to site of delivery; Level III: neonatal intensive care present in same building (in addition to neonatology units) or in immediate proximity to delivery room.

During the study period, the proportions of women aged 35 years or more, BMI ≥ 25 kg/m^2^, and a history of cesarean delivery increased over time (p < 10^–4^) (Table 1). In contrast, the proportions of smokers and women with origins in metropolitan France decreased (p < 10^–4^) (Table 1). Among the instrumental deliveries, the number of deliveries performed under epidural analgesia increased throughout the study period (p < 10^–4^) (Table 2). Over the three phases of the study period, vacuum became the main instrument for deliveries, with the proportion of vacuum-assisted deliveries increasing from 28.5 to 44.3%, whereas the use of forceps decreased from 48 to 28.9% (p < 10^–4^) (Table 2). The proportion of neonates weighing more than 4000 g decreased over time (p < 10^–4^), but there was no significant change in the cranial circumference (p = 0.9) (Table 2). Finally, throughout the study period, women were more inclined to deliver at level 2 (presence of a neonatal department and special care unit in the same building than maternity) or 3 (neonatal intensive care unit in the same building than maternity) compared to level 1 maternity (no neonatal department) (p < 10^–4^) (Table 2).

**Figure 2:**
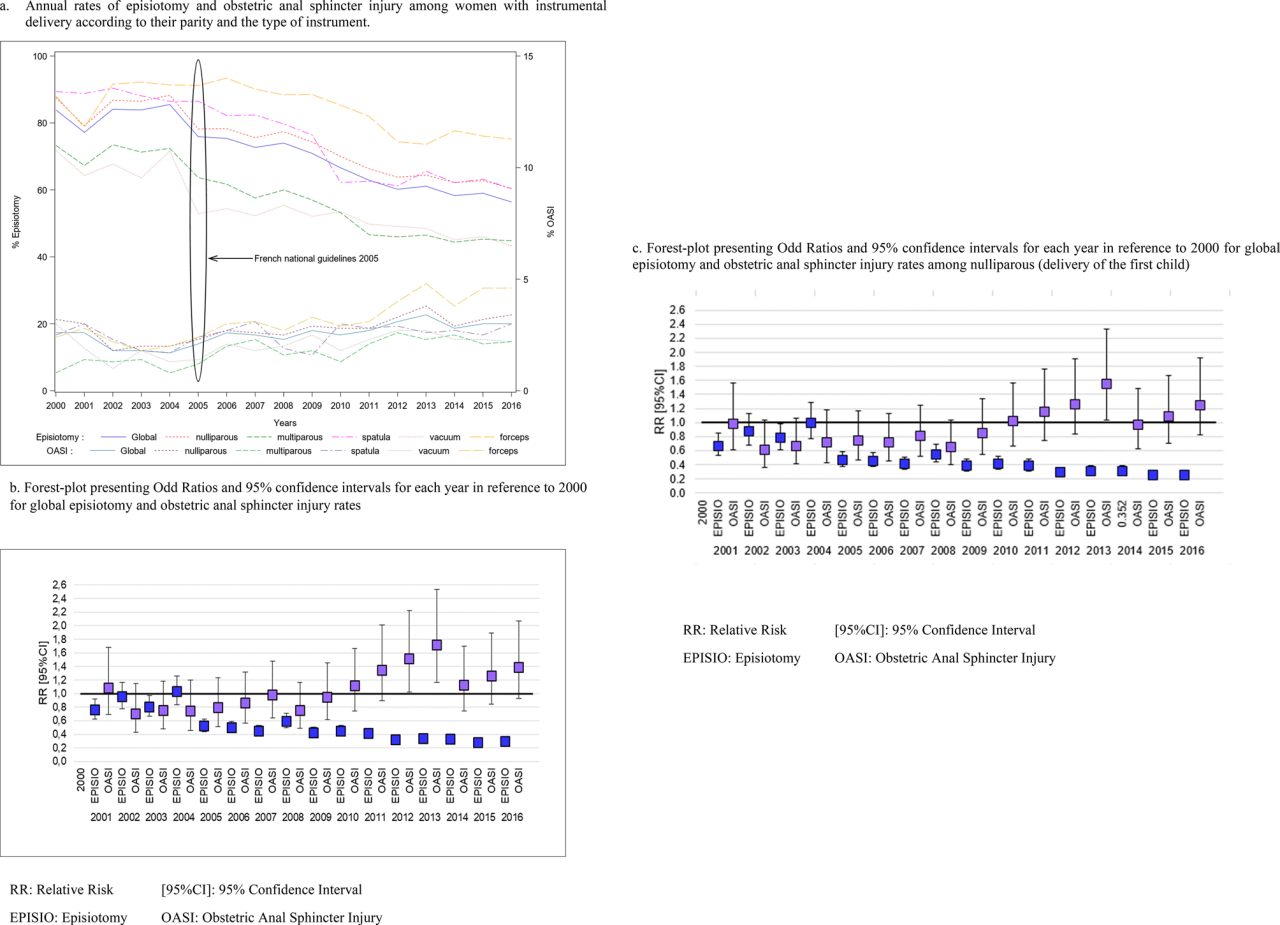
Impact of 2005 French national guidelines on the episiotomy and obstetric anal sphincter injury rates.

The overall rate of episiotomy during instrumental delivery decreased between 2000 (83.9%) and 2016 (56.4%) (Fig. 2a). The range of the reduction was dependent on the parity and type of instrument considered.

Compared to 2000–2005, the risk of having an episiotomy was 0.85 [95% CI 0.85–0.86] in 2006–2011 and 0.73 [95% CI 0.72–0.74] in 2012–2016. After taking into account the geographical origin (France, Southern Europe, North Africa, or other origin vs. Asia vs. Africa), BMI (< 25 vs. ≥ 25 kg/m^2^), parity (nulliparous vs. multiparous), instrumental delivery type (vacuum vs. forceps vs. spatulas), percentile birthweight (≤ 90 vs. > 90), head circumference (< 36 cm vs. ≥ 36 cm), and level of the maternity ward (I vs. II vs. II), the aRR was 0.93 for 2006–2011 [95% CI 0.92–0.95], and 0.89 for 2012–2016 [95% CI 0.87–0.90] (Table 3). All the aRRs for the selected subgroups according to parity and type of instrumental delivery indicated a statistically significant risk reduction for episiotomy, except for the subgroup of multiparous and forceps deliveries in the 2006–2011 period compared to the 2000–2005 period.

**Table 3:** Episiotomy and obstetric anal sphincter injury rates in the overall cohort and according to 3 studied periods.

	Total N = 96,035 % (n)	2000–2005 N = 16,264 % (n)	2006–2011 N = 37,686 % (n)	2012–2016 N = 42,085 % (n)	Crude RR [95% CI]	Adjusted RR [95% CI]
**Episiotomy**						
Global	66.9 (64,227)	81.2 (13,212)	69.4 (26,156)	59.1 (24,859)	0.85 [0.85–0.86]^1^	0.93 [0.92–0.95]^1,3^
					0.73 [0.72–0.74]^2^	0.89 [0.87–0.90]^2,3^
Nulliparous	70.2 (49,856)	83.8 (10,018)	72.7 (20,233)	62.8 (19,605)	0.87 [0.86–0.88]^1^	0.94 [0.92–0.95]^1,4^
					0.75 [0.74–0.76]^2^	0.89 [0.88–0.91]^2,4^
Multiparous	52.9 (10,986)	69.6 (2387)	54.9 (4259)	45.4 (4340)	0.79 [0.77–0.81]^1^	0.98 [0.94–1.01]^1,5^
					0.65 [0.63–0.67]^2^	0.89 [0.85–0.93]^2,5^
For spatula	69.6 (16,702)	87.9 (3364)	70.7 (6304)	62.5 (7034)	0.80 [0.79–0.82]^1^	0.87 [0.85–0.90]^1,5^
					0.71 [0.70–0.72]^2^	0.81 [0.79–0.83]^2,5^
For vacuum	50.9 (19,625)	62.7 (2908)	52.6 (8057)	46.4 (8660)	0.84 [0.82–0.86]^1^	0.89 [0.86–0.92]^1,5^
					0.74 [0.72–0.76]^2^	0.85 [0.82–0.88]^2,5^
For forceps	83.4 (27,900)	89.0 (6940)	87.6 (11,795)	75.2 (9165)	0.98 [0.97–0.99]^1^	1.00 [0.98–1.01]^1,5^
					0.85 [0.83–0.86]^2^	0.95 [0.93–0.96]^2,5^
**Obstetric anal sphincter injury**						
Global	2.7 (2581)	2.0 (333)	2.5 (958)	3.1 (1290)	1.24 [1.10–1.40]^1^	1.30 [1.10–1.53]^1,6^
					1.50 [1.33–1.69]^2^	1.57 [1.33–1.85]^2,6^
Nulliparous	2.9 (2073)	2.3 (277)	2.7 (761)	3.3 (1035)	1.18 [1.03–1.35]^1^	1.21 [1.01–1.45]^1,7^
					1.43 [1.26–1.63]^2^	1.47 [1.23–1.75]^2,7^
Multiparous	2.0 (406)	1.2 (41)	1.8 (142)	2.3 (223)	1.53 [1.08–2.16]^1^	1.98 [1.25–3.14]^1,7^
					1.95 [1.40–2.72]^2^	2.36 [1.50–3.72]^2,7^
For spatula	2.6 (623)	2.2 (84)	2.6 (231)	2.7 (308)	1.18 [0.92–1.51]^1^	1.31 [0.94–1.82]^1,8^
					1.25 [0.98–1.58]^2^	1.47 [1.06–2.05]^2,8^
For vacuum	2.2 (846)	1.6 (75)	2.1 (320)	2.4 (451)	1.29 [1.01–1.66]^1^	1.35 [0.99–1.84]^1,8^
					1.50 [1.17–1.91]^2^	1.63 [1.20–2.21]^2,8^
For forceps	3.3 (1112)	2.2 (174)	3.0 (407)	4.4 (531)	1.35 [1.14–1.61]^1^	1.24 [0.97–1.59]^1,8^
					1.95 [1.65–2.31]^2^	1.65 [1.29–2.12]^2,8^

^1^RR: 2006–2011 vs. 2000–2005.

^2^RR: 2012–2016 vs. 2000–2005.

^3^RR adjusted for: geographical origin (France or Southern Europe or North Africa or other origin vs. Asia vs. Africa), BMI (< 25 vs. ≥ 25), parity (nulliparous vs multiparous), instrumental delivery type (vacuum vs. forceps, vs. spatulas), percentile birthweight (≤ 90 vs. > 90), head circumference (< 36 cm vs. ≥ 36 cm) and level of maternity ward (I vs. II, vs. III).

^4^RR adjusted for: adjusted for: geographical origin (France or Southern Europe or North Africa or other origin vs. Asia vs. Africa), BMI (< 25 vs. ≥ 25), instrumental delivery type (vacuum vs. forceps, vs. spatulas), percentile birthweight (≤ 90 vs. > 90), head circumference (< 36 cm vs. ≥ 36 cm) and level of maternity ward (I vs. II, vs. III).

^5^RR adjusted for: geographical origin (France or Southern Europe or North Africa or other origin vs. Asia vs. Africa), BMI (< 25 vs. ≥ 25), parity (nulliparous vs multiparous), percentile birthweight (≤ 90 vs. > 90), head circumference (< 36 cm vs. ≥ 36 cm) and level of maternity ward (I vs. II, vs. III).

^6^RR adjusted for: geographical origin (France or Southern Europe or North Africa or other origin vs. Asia vs. Africa), BMI (< 25 vs. ≥ 25), parity (nulliparous vs multiparous), instrumental delivery type (vacuum vs. forceps, vs. spatulas), episiotomy (yes, no), percentile birthweight (≤ 90 vs. > 90), head circumference (< 36 cm vs. ≥ 36 cm) and level of maternity ward (I vs. II, vs. III).

^7^RR adjusted for: adjusted for: geographical origin (France or Southern Europe or North Africa or other origin vs. Asia vs. Africa), BMI (< 25 vs. ≥ 25), instrumental delivery type (vacuum vs. forceps, vs. spatulas), episiotomy (yes, no), percentile birthweight (≤ 90 vs. > 90), head circumference (< 36 cm vs. ≥ 36 cm) and level of maternity ward (I vs. II, vs. III).

^8^RR adjusted for: geographical origin (France or Southern Europe or North Africa or other origin vs. Asia vs. Africa), BMI (< 25 vs. ≥ 25), parity (nulliparous vs multiparous), episiotomy (yes, no), percentile birthweight (≤ 90 vs. > 90), head circumference (< 36 cm vs. ≥ 36 cm) and level of maternity ward (I vs. II, vs. III).

The risk of OASI was 1.24 [95% CI 1.10–1.40] in 2006–2011 and 1.50 [95% CI 1.33–1.69] in 2016–2016 compared to 2000–2005. After adjusting for the same confounding factors as those reported above plus episiotomy (yes/no), the aRR was 1.30 [95% CI 1.10–1.53] in 2006–2011 and 1.57 [95% CI 1.33–1.85] in 2012–2016 (Table 3). All the aRRs for OASI occurrence indicated an increased risk for OASI except those for the spatula, vacuum, and forceps groups, for whom the difference was statistically significant only when comparing 2012–2016 to 2000–2005 (Table 3). The risks for the occurrence of 3rd or 4th degree perineal tears are available in Table 4.

**Table 4:** Third- and fourth-degree perineal tears rates in the overall cohort and according to the 3 studied periods.

	Total N = 96,035% (n)	2000–2005 N = 16,264% (n)	2006–2011 N = 37,686 % (n)	2012–2016 N = 42,085 % (n)	Crude RR [95% CI]	Adjusted RR [95% CI]
**3rd degree**^**6**^						
Global	2.4 (2333)	1.7 (278)	2.3 (854)	2.9 (1201)	1.33 [1.16–1.52]^1^	1.38 [1.15–1.65]^1,3^
					1.67 [1.47–1.90]^2^	1.72 [1.44–2.05]^2,3^
Nulliparous	2.6 (1876)	2.0 (234)	2.4 (676)	3.1 (966)	1.24 [1.07–1.44]^1^	1.29 [1.06–1.57]^1,4^
					1.58 [1.37–1.82]^2^	1.63 [1.34–1.97]^2,4^
Multiparous	1.8 (369)	1.0 (33)	1.7 (130)	2.2 (206)	1.74 [1.19–2.55]^1^	2.01 [1.24–3.25]^1,4^
					2.24 [1.55–3.23]^2^	2.34 [1.46–3.77]^2,4^
For spatula	2.4 (567)	2.0 (75)	2.3 (204)	2.6 (288)	1.17 [0.90–1.52]^1^	1.25 [0.88–1.77]^1,5^
					1.31 [1.01–1.68]^2^	1.47 [1.04–2.08]^2,5^
For vacuum	2.0 (786)	1.4 (63)	1.9 (292)	2.3 (431)	1.41 [1.07–1.84]^1^	1.45 [1.03–2.02]^1,5^
					1.70 [1.31–2.21]^2^	1.80 [1.29–2.51]^2,5^
For forceps	2.9 (980)	1.8 (140)	2.7 (358)	4.0 (482)	1.48 [1.22–1.80)^1^	1.40 [1.07–1.84]^1,5^
					2.20 [1.83–2.65]^2^	1.89 [1.44–2.49]^2,5^
**4th degree**^**7**^						
Global	0.3 (248)	0.3 (55)	0.3 (104)	0.2 (89)	0.82 [0.59–1.13]^1^	0.86 [0.54–1.37]^1,3^
					0.63 [0.45–0.87]^2^	0.79 [0.48–1.28]^2,3^
Nulliparous	0.3 (197)	0.4 (43)	0.3 (85)	0.2 (69)	0.85 [0.59–1.22]^1^	0.81 [0.50–1.33]^1,4^
					0.61 [0.42–0.90]^2^	0.67 [0.39–1.13]^2,4^
Multiparous	0.2 (37)	0.2 (8)	0.2 (12)	0.2 (17)	0.66 [0.27–1.62]^1^	1.62 [0.31–8.55]^1,4^
					0.76 [0.33–1.77]^2^	2.67 [0.54–13.24]^2,4^
For spatula	0.2 (56)	0.2 (9)	0.3 (27)	0.2 (20)	1.29 [0.61–2.73]^1^	2.01 [0.65–6.24]^1,5^
					0.76 [0.34–1.66]^2^	1.42 [0.44–4.66]^2,5^
For vacuum	0.2 (60)	0.3 (12)	0.2 (28)	0.1 (20)	0.71 [0.36–1.39]^1^	0.87 [0.38–2.02]^1,5^
					0.41 [0.20–0.85]^2^	0.75 [0.31–1.80]^2,5^
For forceps	0.4 (132)	0.4 (34)	0.4 (49)	0.4 (49)	0.83 [0.54–1.29]^1^	0.57 [0.28–1.17]^1,5^
					0.92 [0.60–1.43]^2^	0.68 [0.33–1.40]^2,5^

^1^RR: 2006–11 vs. 2000–2005.

^2^RR: 2012–16 vs. 2000–2005.

^3^RR 2006–11 vs. 2000–2005 adjusted for: geographical origin (France or Southern Europe or North Africa or other origin vs. Asia vs. Africa), BMI (< 25 vs. ≥ 25), parity (nulliparous vs multiparous), instrumental delivery type (vacuum vs. forceps, vs. spatulas), episiotomy (yes, no), percentile birthweight (≤ 90 vs. > 90), head circumference (< 36 cm vs. ≥ 36 cm) and level of maternity ward (I vs. II, vs. III).

^4^RR 2006–11 vs. 2000–2005 adjusted for: adjusted for: geographical origin (France or Southern Europe or North Africa or other origin vs. Asia vs. Africa), BMI (< 25 vs. ≥ 25), instrumental delivery type (vacuum vs. forceps, vs. spatulas), episiotomy (yes, no), percentile birthweight (≤ 90 vs. > 90), head circumference (< 36 cm vs. ≥ 36 cm) and level of maternity ward (I vs. II, vs. III).

^5^RR 2006–11 vs. 2000–2005 adjusted for: geographical origin (France or Southern Europe or North Africa or other origin vs. Asia vs. Africa), BMI (< 25 vs. ≥ 25), parity (nulliparous vs multiparous), episiotomy (yes, no), percentile birthweight (≤ 90 vs. > 90), head circumference (< 36 cm vs. ≥ 36 cm) and level of maternity ward (I vs. II, vs. III).

^6^Third-degree tears involve the anal sphincter, with either total or partial damage to the sphincter.

^7^Fourth-degree tears involve the anal sphincter and tears into the rectal mucosa.

Significant variations in episiotomy rates were highlighted throughout the study period (from 2000 to 2016) by using univariate and multivariate Prais–Winsten regression (p < 0.001). We observed a bearing rate between 2000 and 2004, followed by a continuous reduction in the episiotomy rate until 2016 (Fig. 2b). The OASI rate showed a significant decrease from 2000 to 2016 (p = 0.038), but the multivariate analysis failed to show a statistically significant difference (p = 0.10) (Fig. 2b). The same result is reported when considering the subgroup of nulliparous women (Fig. 2c). We did not find any interactions in the multivariate analyses.

## Discussion

### Principal findings

After the publication of the 2005 French guidelines in favor of the more restricted use of episiotomy, the global rate of episiotomy in instrumental delivery decreased from 81.2 to 59.1%. During the same period, the rate of OASI in case of instrumental delivery increased from 2 to 3.1%. The risk of undergoing an episiotomy was lower in 2006–2011 (aRR 0.93 [95% CI 0.92–0.95]) and 2016–2016 (aRR 0.89 [95% CI 0.87–0.90]), while the risk of OASI was higher in 2006–2011 (aRR 1.30 [95% CI 1.10–1.53] and 2012–2016 (aRR 1.57 [95% CI 1.33–1.85]), in comparison with the 5-year period before the publication of the 2005 guidelines (2000–2005). However, we did not find a difference in the OASI risk in the Prais–Winsten regression.

### Results in the context of what is known

Our findings showed a reduction in the episiotomy rate following the 2005 French guidelines, but the OASI increase occurrence did not reach a significant difference in the Prais–Winsten regression. This seems discordant with the findings of a previous monocentric study reporting a drastic reduction from 78 to 16.8% in episiotomy use and an increase from 3.1 to 12.7% in OASI occurrence between 2005 and 2016^12^. That study reported a continuous evolution, and the lower the episiotomy rate, the higher was the OASI rate^12^. It is likely that in the present study, we failed to report a significant increase in OASI occurrence for the whole period because the decrease in episiotomy use was less substantial than that reported in the previous monocentric study^12^. This is also supported by our subgroup’s analysis of the type of instrument, which showed that the increase in the risk of OASI occurrence became statistically significant only when comparing the last period (2012–2016), when the episiotomy rate was the lowest, to the reference one, for all types of instruments. We observed a decrease in OASI occurrence after 2014 but without any chronological association with any French updated guidelines. Last French guidelines about perineal prevention at childbirth had been published in 2018 and there was no change in the classification of perineal tears^1^. One explanation could be, in part, a variation of factors linked to an increase or reduction of perineal tears (more important use of vacuum instead of forceps delivery, increase of maternal age or epidural analgesia during the last years of the study) (Tables 1, 2). Although the objective of Laine et al. study differed from ours, their results are interesting. In this historical cohort study comparing two study periods (2003–2005 and 2008–2010, n = 31,709 women and 907 sphincter injuries), the occurrence of OASI before and after implementing a training program for improve perineal support at childbirth was reduced from 4 to 1.9%^23^. This OASI rate was reduced in case of vacuum delivery among the two periods (10.8% vs 5%). When adjusted for risk factors in the multivariate analysis, episiotomy appeared as a protective factor for OASIS in both time periods for primiparous women^23^. A weakness of the Laine et al. study is that the use of perineum support method, if used during second stage of delivery, was not registered in the medical records, and therefore, use of perineum support could not be assessed directly in this retrospective study. In France, most midwives preferred the hands-on technique at childbirth (91.4%)^24^.

### Clinical implications

The effect of decreasing the use of episiotomy during instrumental delivery on OASI occurrence might have been attenuated by changes in the modalities used for instrumental deliveries. Indeed, we reported a significant decrease in the use of forceps (from 48 to 28.9%) and a corresponding increase in the use of vacuum delivery (from 28.5 to 44.3%), which became the most frequently used instrumental technique. There is a high level of evidence showing that the risk of OASI is more important when the delivery is performed using forceps, an instrument that increases the diameter of cephalic presentation, than during vacuum-assisted deliveries^1,5,25^. In addition, we observed a change in the neonate characteristics, with a significant decrease in the proportion of neonates large for gestational age at birth (from 9.3 to 7.6%), which is an important risk factor for OASI. Another factor that may have attenuated the effect of restricting the use of episiotomy on OASI occurrence is the reduction in the proportion of nulliparous women, which is the most important risk factor reported in the literature^1,2,5^. One interpretation of our results that could be important in clinical practices is that when birth attendants are instructed to use episiotomy more restrictively, they will probably use it when the risk of OASI is estimated as very high. This could be a reason for the absence of statistical association between the decrease in episiotomy rate and the increase in OASI occurrence. One point that could sustain this hypothesis is that in the Jiang et al. Cochrane review, about spontaneous delivery, episiotomy was not protective from OASI occurrence and there is even an increase in OASI occurrence in case of episiotomy with the hypothesis that most of high-risk cases were in the episiotomy group^7^.

### Research implications

Finally, with respect to the changes observed during the overall study period, even if we did not report a significant increase in OASI occurrence, this does not invalidate the hypothesis of an association between a low episiotomy rate and a high OASI rate during instrumental delivery. The findings instead highlights the limitations of considering available international retrospective studies for assessing the effect of episiotomy in this indication and explain the low level of evidence in international guidelines^1,2,25^. We still need a high-level evidence data, but a randomized trial appears very difficult to implement in this situation for practical (high number of women to randomize in the labor ward) and methodological reasons such as contamination of methods in different study arms and blinding of patients or care providers. Murphy et al. reported the experience of a pilot randomized trial comparing routine versus restrictive episiotomy during instrumental delivery^9^. This study highlighted some difficulties that would be very difficult to avoid, as an example for women randomized in the restrictive group in case of forceps delivery the rate of episiotomy was 64.2%. Even considering these difficulties, the authors considered that an RCT is feasible but will require a large sample size. They suggested the inclusion of 1600 women leading to screen 8000 women in antenatal period^9^. Such a randomized trial has not been started yet whereas this pilot study was published in 2008. However, there is currently an attempt of a randomized trial comparing lateral versus no episiotomy to reduce OASI’s occurrence in case of vacuum delivery in nulliparous women^26^. Furthermore, randomized trials are not the best methodological scheme for investigating complex interventions such as episiotomy and instrumental delivery that depend on the context, organization, population, and environmental factors^27,28^. Therefore, a large prospective national observational study appears to be the best approach to study this question. This study is currently ongoing in France in 120 maternity with 15,000 women planned to include (INSTRUMODA; NCT:04446780; https://clinicaltrials.gov)^29^.

### Strengths and limitations

The first strength of this study is that we reported data from a national multicentric cohort of about 96,035 deliveries in a country (France) with a strong culture in favor of vaginal delivery and the performance of obstetrical maneuvers^16–18,30^. In 2016, the rates of instrumental delivery and cesarean delivery were 12.2% and 20.4%, respectively^6^. Another strength is that we aimed to report an analysis using a dynamic approach by investigating the effect of our national guidelines on the rates of episiotomy and OASI. Most of the existing studies used data from national administrative registers to evaluate the association between episiotomy and OASI occurrence without considering the potential changes in episiotomy use across time^8,10,11,31^. Thus, the analysis in this study represents an innovative approach.

A first limitation is that, as reported above, the maternity wards choose one month to participate however, with the computerization of medical files of the maternity wards in France, more and more maternity wards send the entire data years to the national database. Accepted only the same month for all maternity wards would induce a seasonal bias because of the calendar variation of several diseases. Second, our multivariate models did not account for the type of fetal head orientation (anterior or posterior occiput), which is a factor associated with OASI^1,2^. The AUDIPOG database contained limited data for this outcome. Nevertheless, we considered that this did not bias our analysis because there are no findings suggesting that the proportion of deliveries with fetuses in posterior occiput orientation would have changed over time. Another point is that it is likely that changes in episiotomy rates were heterogeneous among the French maternity wards. For example, in our unicentric experience we reported a massive decrease from 78 to 16% which is probably not the case for all French maternities. Regarding the high number of maternity wards considered in this study we can consider that this is not a bias and that the tendance observed in this study is representative of the national tendance. It is also likely that the risk of OASI depend of the magnitude of the change in episiotomy rate supporting the importance of our analysis considering three consecutives periods. Indeed, we’ve reported that the most important increased risk of OASI was observed when the rate of episiotomy was the lowest whereas it did not reach significance for some subgroups (forceps for example) during the first years after the publication of the guidelines. Upcoming prospective studies will have this challenge to try identifying the reasonable rate of episiotomy during instrumental delivery, meaning to identify high risk women. Another limitation was that we were unable to report the indication and type of episiotomy from the AUDIPOG database. Here again, we considered that this factor did not bias our findings since French and international guidelines recommend using a mediolateral incision when an episiotomy is decided and the French national guidelines did not stress a formal indication for episiotomy^1,2,13^. Furthermore, a recent national survey reported that the rate of usage of midline episiotomy was lower than 0.5% among French obstetricians^32^. Finally, our study did not provide data about the angle of episiotomy. Even if we can consider that almost all episiotomies were mediolateral ones we are not able to control the angle of section. This is a limitation because it is reported that there is an important difference between the self-assessment of the section angle by the obstetrician and the effective angle measured on the perineum with a significant rate of episiotomy being at less than 45° from the midline. This is very important regarding hath all international guidelines recommend a minimal angle of 45° measured on the perineum (meaning a minimal section angle at 60°)^1,2,33^. This limitation is inherent to retrospective studies but it is crucial that future prospective studies control this bias.

## Conclusion

After the publication of the 2005 French guidelines in favor of more restricted use of episiotomy, we observed a significant decrease in episiotomy rate. During the same period we observed an increase in the crude rate of OASI but with a less important extent and which did not reach statistical significance in multivariate Prais–Winsten regression analysis. Several changes in the characteristics of women and neonates, as well as in instrumental delivery modalities, might have attenuated the effect of reduced episiotomy use on the risk of OASI in this context. Further prospective population-based studies are required to assess the role of episiotomy in OASI occurrence, and its prevention, during instrumental delivery.
